# Systematics of the seed beetle genus *Decellebruchus* Borowiec, 1987 (Coleoptera, Bruchidae)

**DOI:** 10.3897/zookeys.579.7716

**Published:** 2016-04-11

**Authors:** Jesús Romero Nápoles

**Affiliations:** 1 Especialidad de Entomología y Acarología, Postgrado en Fitosanidad, Colegio de Postgraduados, Montecillo, Estado de México, MEXICO

**Keywords:** Seed beetles, Decellebruchus, Bruchidae, cladistics

## Abstract

Keys to species, descriptions, synonymy, host plants, and geographical distributions are presented for the three species in the genus *Decellebruchus* (Borowiec 1987); of those, *Decellebruchus walker* (Pic 1912) was the only species included at the time of the genus denomination, *Decellebruchus atrolineatus* (Pic 1921) is transferred to this genus, and *Decellebruchus lunae* is described as a new species. The shortest and most parsimonious phylogenetic tree for genera with pectinate antennae had a length of 33, consistency index 87, and retention index 81.

## Introduction

The monotypic genus *Decellebruchus* was erected by Borowiec in 1987 with the type species *Decellebruchus walkeri* (Pic 1912). This species has a complicated history, starting with *Bruchus figuratus* that was described by Walker (1859); however this was a homonym and then Pic (1912) proposed the replacement name of *Bruchus walkeri*. Later Decelle (1975b) transferred it to *Bruchidius*, but because the unusual pectinate antennae of the species in the genus *Bruchidius*, Borowiec (1987) erected the monotypic genus *Decellebruchus* for it. Another species that has been included unofficially in the latter genus is *Bruchus atrolineatus* Pic, 1921; however *Bruchus atrolineatus* has also suffered a series of generic changes through time. Finally the third species included in the genus is the new species *Decellebruchus lunae*. In order to clarify the genus it is reviewed and a hypothesis of its phylogeny is proposed.

## Material and methods

**Specimens.** Preparation of genitalia for study followed the techniques and nomenclature described by Kingsolver (1970) and modified by Romero and Johnson (1999). For specimens the following collections were consulted: Colección Entomológica del Colegio de Postgraduados, Montecillo, Estado de México, México (CEAM), Florida State Collection of Arthropods, Gainesville, Fl, USA (FSCA), Musee Zoologique de l’Universite de Lund, Lund University, Sweden (MZLU); South African National Collection of Insects, Queenswood, South Africa (SANC).

**Cladistics.** External morphological characters and internal characters, these latter only from male genitalia (Table 1) were used. Host plants and distribution were also considered. Taxa included in the analysis were only those bruchids with pectinate antennae (excluding the genera *Caryedes* and *Gibbobruchus* because they have only few species with pectinate antennae) and all species in the genus *Decellebruchus*. *Pachymerus*, a less derived genus was used as the outgroup. The data matrix is presented in Table 2. The program Hennig86 (Farris 1988) was used to generate the cladogram, although a comparative tree was obtained with Mesquite, version 3.04 (Maddison and Maddison 2015) using the same data matrix.

**Table 1.** Characters used for the cladistic analysis for Bruchidae with pectinate antennae.

**External morphology**	
0. Body length	8–16 mm: 0; 1.5–5.4 mm: 1
1. Eye sinus	Shallow: 0; deep: 1
2. Male antenna shape	Not pectinate: 0; pectinate 1
3. Last antennal segment	About half as long as hind tibia: 0; as long as or longer than hind tibia: 1
4. Carina media on frons	With carina: 0; without carina: 1
5.Pronotal carina	Complete: 0; no complete:1
6. Pronotum shape	Not conical, without distinctly concave sides: 0; conical with distinctly concave sides: 1
7. Pronotal disc	Without gibbosites: 0; with gibbosites: 1
8. Scutellum shape	Subsquare: 0; elongate: 1
9. Metepisternal sulcus	With sulcus:0; without sulcus: 1
10. Tenth elytral stria	Extending nearly to apex of elytron: 0; extending to half of elytron: 1
11. Elytral basal tubercles	Without tubercles: 0; with tubercles: 1
12. Pygidium articulation	Pygidium and penultimate tergum partially fused and no covered by elytra 1; Penultimate tergum no fused to pygidium and covered by elytra 2
13. Hind femur	Without carinae: 0; with one carina or obsolete carinae 1; bicarinate: 2
14. Spines on hind femur	With 10-14 spines: 0; with 1 to 3 spines: 1; without spines: 2
15. Hind tibia carinae	With complete set of carinae: 0; incomplete set of carinae: 1
16. Tuft of white setae on fore coxa	Without tuft of white setae: 0; with tuft of white setae: 1
17. Male pygidium	Reclinate 0; vertical 1
**Internal morphology**	
18. Lateral lobes of male genitalia	Fused 0; divided 1
19. Ventral valve of male genitalia	No arcuate: 0; deeply arcuate 1
20. Shape of apical portion of median lobe of male genitalia|	No bulbous: 0; bulbous: 1
21. Basal strut of lateral lobes of male genitalia	With an obsolete or small perpendicular keel: 0; with strong perpendicular keel 1
22. Armature of internal sac of male genitalia	Only with spinules: 0; with spinules and small teeth 1
**Ecological characters**	
23. Distribution	Worldwide 0; New world 1; Old world: 2
24. Host plants	Arecaceae: 0; Fabaceae: 1; Convolvulaceae: 2

**Table 2.** Data matrix.

**Taxa**	**Character**																								
	0	1	2	3	4	5	6	7	8	9	10	11	12	13	14	15	16	17	18	19	20	21	22	23	24
1. *Pachymerus*	0	0	0	0	1	0	0	0	0	0	0	0	0	0	0	0	0	0	0	0	0	0	0	1	0
2. *Kytorhinus*	1	1	1	1	0	1	0	0	1	1	0	0	1	1	2	1	0	1	0	0	0	0	0	0	1
3. *Megacerus*	1	1	1	1	1	1	0	0	0	1	1	0	0	1	1	0	0	1	0	0	0	0	0	1	2
4. *Callosobruchus*	1	1	1	1	1	1	0	1	0	1	0	1	0	2	1	0	0	1	1	0	0	0	0	2	1
5. *Conicobruchus*	1	1	1	1	1	1	1	0	0	1	0	0	0	1	1	1	0	0	1	0	0	0	0	2	1
6. *Rhipibruchus*	1	1	1	1	1	1	0	1	0	1	0	1	0	1	1	0	0	1	1	0	0	0	0	1	1
7. *Pectinibruchus*	1	1	1	1	1	1	0	1	1	1	0	0	0	1	1	1	0	1	1	0	0	0	0	1	1
8. *Decellebruchus atrolineatus*	1	1	1	1	1	1	0	1	0	1	0	1	0	1	1	0	1	1	1	1	0	0	0	2	1
9. *Decellebruchus walkeri*	1	1	1	1	1	1	0	1	0	1	0	1	0	1	1	0	1	1	1	1	1	0	1	2	1
10. *Decellebruchus lunae*	1	1	1	1	1	1	0	1	0	1	0	1	0	1	1	0	1	1	1	1	0	1	1	2	1

## Results and discussion

### Key to genera of Bruchidae with pectinate antennae.

**Table:** 

1	Pygidium with one or two tergites exposed behind elytra; antenae in males pectinate or strongly serrate, in female serrate	***Kytorhinus* Bridwell**
–	Pygidium covered at base by elytra	**2**
2	Tenth elytral stria shortened, extending to middle of elytron; antennae in males pectinate, in female serrate	***Megacerus* Fahraeus**
–	Tenth elytral stria extending nearly to apex of elytron	**3**
3	Hind femur bicarinate, with spine on both internal and external ventral margins; antennae in males strongly serrate or pectinate, in female subserrate or serrate	***Callosobruchus* Pic**
–	Hind femur not bicarinate or carinae obsolete, or only ventral carina present	**4**
4	Pronotum conical with distinctly concave sides, antennae in males serrate or pectinate, in female subserrate	***Conicobruchus* Decelle**
–	Pronotum not conical, without distinctly concave sides	**5**
5	Last antennal segment as long as or longer than hind tibia	**6**
–	Last antennal segment about half as longer as hind tibia	***Decellebruchus* Borowiec**
6	Hind tibia carinate, mucro longer than lateral coronal denticle; scutellum subcuadrate	***Rhipibruchus* Bridwell**
–	Hind tibia with obsolete lateral carina, mucro absent; scutellum elongate	***Pectinibruchus* Kingsolver**

### Key to species of *Decellebruchus*

**Table:** 

1	Hind femur with only 1 subapical spine	**2**
–	Hind femur with 1 subapical spine followed by 2 smaller spines	***Decellebruchus walkeri* (Pic)**
2	Pygidium with basal spot of white pubescence; antennomere VII 1.76-2.0X wider that longer; hind femur with subapical acuminate spine about as long as width of tibial base	***Decellebruchus lunae* Romero, sp. n.**
–	Pygidium with two pubescent spots apically; antennomere VII 4.4-5.6X wider than longer; hind femur with small subapical acuminate spine about half as long as width of tibial base	***Decellebruchus atrolineatus* (Pic)**

## Taxonomy

### Decellebruchus

Taxon classificationAnimaliaColeopteraBruchidae

Borowiec

Decellebruchus Borowiec, 1987: 149.

#### Description.

**Male. Vestiture**: Moderately dense or dense, variegated; front coxa with a wide tuft of white setae. Body oval and stout. **Head**: Short, strongly constricted behind eyes, postocular lobe very short; eyes bulging, deeply emarginate; frons narrow, with sharp median carina; antennae pectinate from 4^th^ or 5^th^ segment. **Prothorax**: Pronotum subconical, without lateral carina; disc convex, slightly gibbous before scutellum and with shallow median channel; prosternal process narrow, triangular, acute. **Meso- and metathorax**: Scutellum square, bidentate apically; elytral striae regular; striae 4 and 5 abbreviated basally by tubercle. **Legs**: Metacoxa densely punctate; hind femur moderately swollen, ventral carinae obsolete. Internal ventral margin with small subapical spine, often followed by two smaller spines; hind tibia straight, enlarged, with complete or incomplete set of carinae, mucro longer than lateral coronal denticle. **Abdomen**: More or less telescoped, fifth sternite deeply emarginated; pygidium vertical. **Genitalia**: Internal sac of male genitalia lined with fine spines with or without sclerites; ventral valve deeply arcuate. **Female.** Similar to male, except antenna not pectinate, eyes less bulging, pygidium subvertical, last abdominal sternite not emarginated.

### Decellebruchus atrolineatus

Taxon classificationAnimaliaColeopteraBruchidae

(Pic, 1921) comb. n.

Bruchus atrolineatus Pic, 1921: 15, 1932: 36; Decelle 1951: 184, 1961: 8, 1975a: 21 (comb. n.).
Bruchidius atrolineatus : Prevett 1961: 636, 1967: 5, 1971: 247; Booker 1967: 2; Luca 1968a: 188, 1968b: 589; Decelle 1975a: 15; Southgate 1978: 219; Biemont et al. 1982: 2610; Hamon et al. 1982: 327; Pfaffenberger 1985: 3; Germain et al. 1987: 157; Monge et al. 1988: 297, 1989: 95; Huignard et al. 1989: 197; Pouzat and Nammour 1989: 319; Udayagiri and Wadhi 1989: 119; Lenga et al. 1990: 79; Glitho and Huignard 1990: 195; Pfaffenberger and Monge 1991: 309; Pichard et al. 1991: 185; Shimada and Ishihara 1991: 289; Credland 1992: 1; Ishihara and Shimada 1995: 127; Ofuya and Credland 1996: 323; Kingsolver 1988; Sanon et al. 2005; Löbl and Smetana 2010: 341.
Callosobruchus atrolineatus : Zacher 1952: 465; Shomar 1963: 178.
Bruchus semiflabellatus Pic, 1931; Bondar 1936: 37 (syn.); Kingsolver and Silva 1991: 413 (syn.); Udayagiri and Wadhi 1989: 119.
Acanthoscelides semiflabellatus : Blackwelder 1946: 761.
Callosobruchus semiflabellatus : Zacher 1952: 465.
Decellebruchus atrolineatus : Anton 1994: 100; Kergoat et al. 2005: 605 (without indicating clearly new combination).

#### Description.

**Figure 1.**
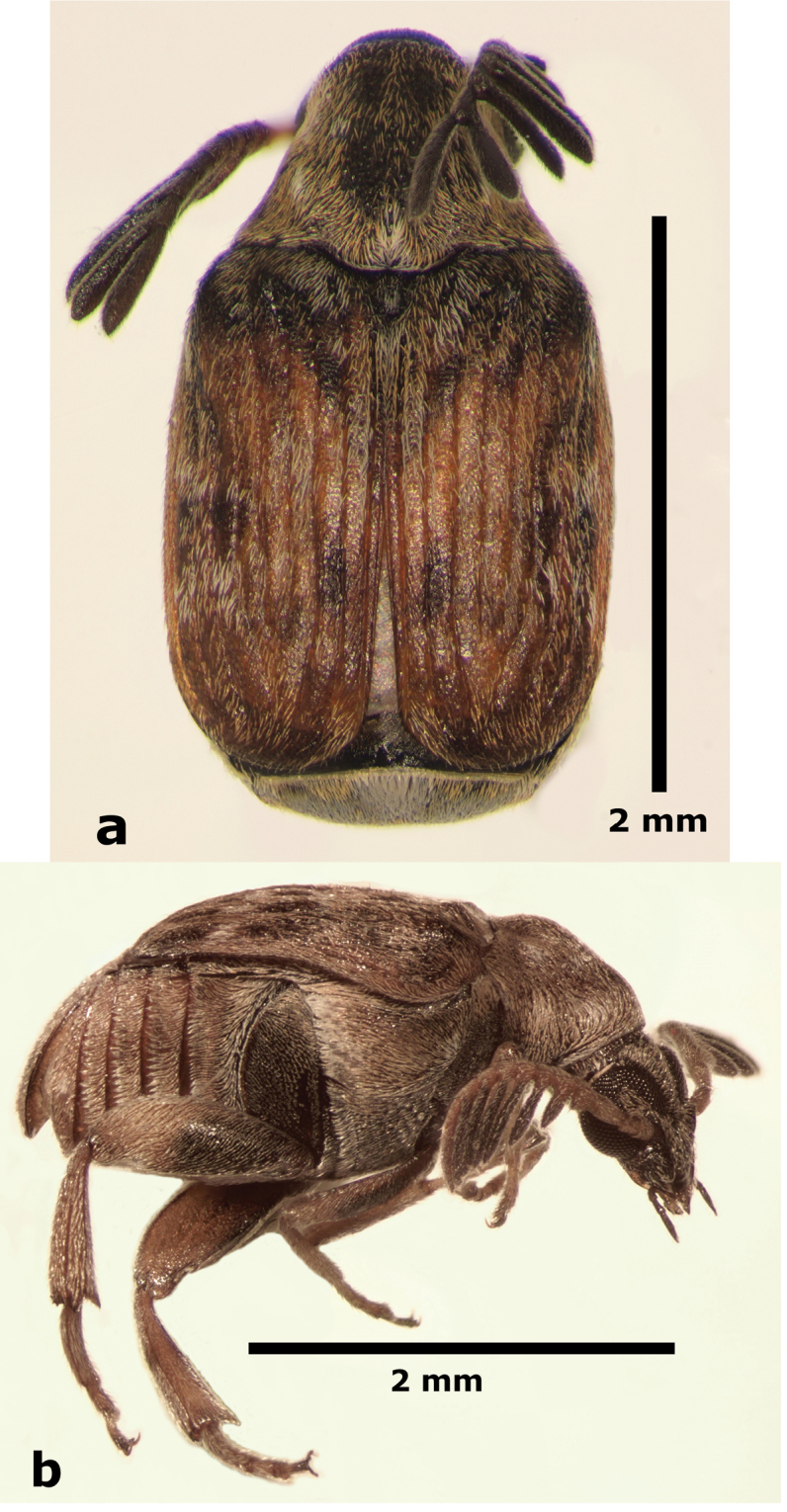
Male habitus of *Decellebruchus atrolineatus*; **a** dorsal view **b** lateral view.

**Figure 2.**
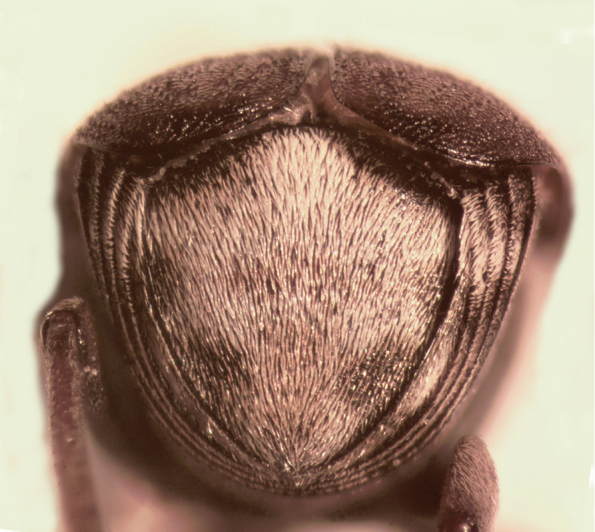
Male pygidium of *Decellebruchus atrolineatus*.

**Figure 3.**
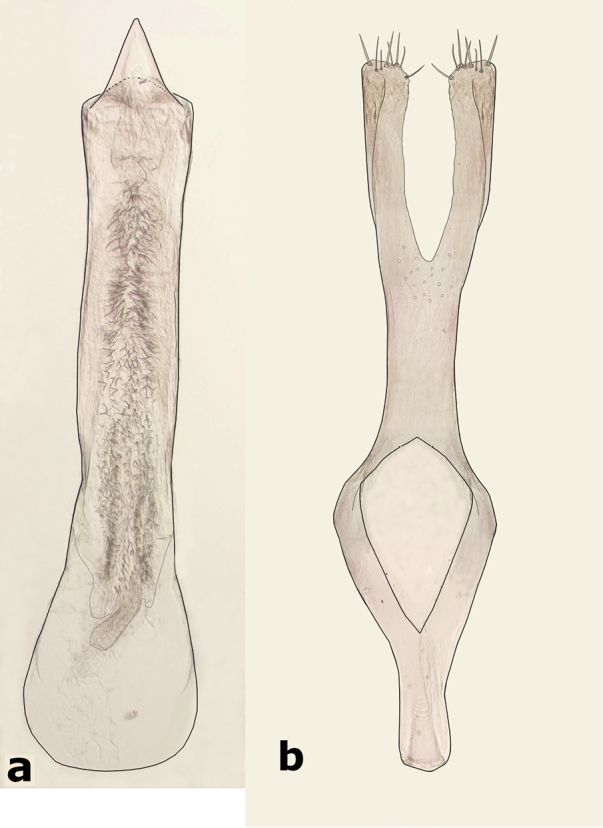
Male genitalia of *Decellebruchus atrolineatus*; **a** median lobe **b** lateral lobes.

**Figure 4.**
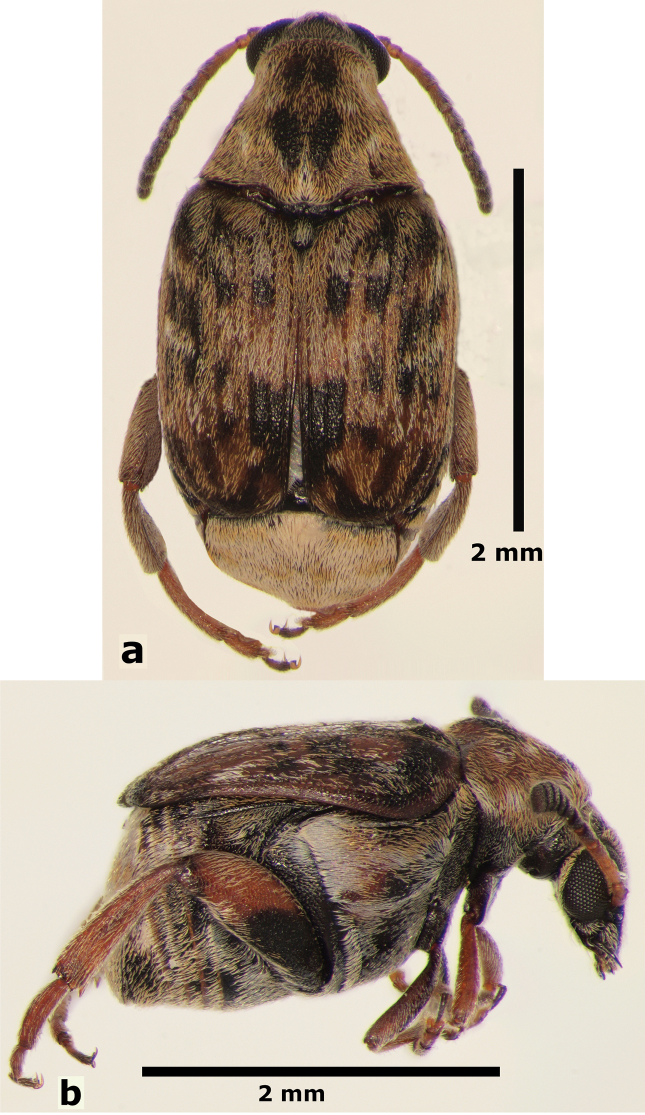
Female habitus of *Decellebruchus atrolineatus*; **a** dorsal view **b** lateral view.

**Male** (Fig. 1a–b). Length (pronotum-elytra): 2.4–2.6 mm; width: 1.4–1.6 mm; maximum thoracic depth 1.5–1.7 mm. **Color**: Antennae with the first three segments yellowish, the rest dark or partially dark; head, prosternum, metasternum, base of meso-femur and meta-femora, and coxae dark; pronotum with two longitudinal dark bands, which may together form a dark spot; elyton variegate; pygidium with three pairs of dark spots, two apical, two median-lateral, and two basal; rest of body yellowish. **Vestiture**: Body with mixed yellowish and white pubescence; scutellum with whitish pubescence; fore coxa with a tuft of white setae; pygidium with yellowish and whitish pubescence forming a variegate pattern. **Head**: Short and broad, densely micropunctulate, frons with a strong median carina, distance between eyes 2.3.–3.3× as wide as eye width, eye cleft 0.60–0.71× its length by ocular sinus, posterior margin of eye protruding from adjacent surfaces, postocular lobe rounded and setose; distance from base of antennae to apex of labrum 0.39–0.55× as long as distance from upper limits of eyes to apex of labrum; antennomeres I–III filiform, IV subserrate, V–XI pectinate; antennomere II 2.8–3.8× as long as antennomere XI; antennomere VII 4.4–5.6× wider that long; antenna extending slightly beyond humerus. **Prothorax**: Subconical, without lateral carina; densely foveolate, disc convex, slightly gibbous before scutellum and with shallow median channel; prosternal process narrow, triangular, acute, half as long as procoxae. **Meso- and metathorax**: Scutellum square, bidentate apically; elytral striae regular, striae 4 and 5 abbreviated basally by tubercle, humeri raised. **Legs**: First protarsomere 2.0–3.0× as long as second, first mesotarsomere 1.8–2.3× as long as second, first metatarsomere 2.6–3.0× as long as second; metacoxa densely punctate; hind femur constricted basally and apically, expanded medially to about width of coxa; without external carina ventrally; internal ventral carina with small subapical acuminate spine about half as long as width of tibial base; hind tibia straight, enlarged, with only mesal and ventral carinae; tibial corona with 4 spinules, mucro 0.10–0.13× as long as first tarsomere; without sinus at base of spine; first hind tarsomere with ventrolateral glabrous longitudinal carina. **Abdomen**: Pygidium vertical (Fig. 2); last sternite emarginate. **Genitalia**: Median lobe moderately long, ventral valve triangular and deeply arcuate, internal sac with many small spines or needles, without large sclerites (Fig. 3a); lateral lobes elongate, expanded at apex, cleft about 0.53 their length; basal strut an obsolete perpendicular keel (Fig. 3b). **Female** (Fig. 4a–b). Length (pronotum-elytra) 2.4–2.8 mm, width: 1.5–1.7 mm, Maximum thoracic depth 1.6–1.8 mm. Similar to male except antennae serrate; distance between eyes 1.8.–2.0× as wide as eye width; pygidium subvertical; last sternite not emarginate.

#### Material examined.

**NAMIBIA**: Rundu, 28/V/2015, T. Chauke, 17°55’S 19°46’E, reared seed *Glycine max* (L.), intercepted at Pretoria SAAFQIS Plant Quarantine Station, South Africa, Sample Pta. 2811 (1 ex SANC). Caprivi region, 2002, intercepted at Pretoria SAAFQIS Plant Quarantine Station, South Africa (70 ex SANC). **AFRICA**: Intercepted at USA, 36/XII/2003, reared seed *Phaseolus* sp. (3 ex, FSCA); Intercepted at Atlanta, USA, 10/IX/2006, reared seed *Phaseolus vulgaris* L. (1 ex., FSCA). **DEMOCRATIC REPUBLIC OF THE CONGO**: N of Shaba Province, 28/III/1980, Whitecomb W.H., in cowpeas (1 ex., FSCA). NIGERIA: Intercepted at USA, 2/II/2004, *Phaseolus* sp. (7 ex., FSCA); Intercepted at USA, 3/II/2004, reared seed *Phaseolus* sp. (2 ex., CEAM). **MEXICO**: Ocolome, El Fuerte, Sinaloa, 21/I/2013, Lugo G.G.A., reared seed *Vigna unguiculata* (L.) WALP. (190 ex., CEAM).

#### Host plants.

*Dolichos lablab* L., *Glycine max* (L.), *Phaseolus vulgaris* L. *Vigna unguiculata* (L.) Walp., Vigna unguiculata subsp. stenophylla (Harv.) Maréchal & Al., Vigna unguiculata subsp. unguiculata (L.)Walp. (Fabaceae). Zacher (1952) stated that *Lablab niger* Medik. and *Medicago sativa* L. are plant hosts of *Decellebruchus atrolineatus*, however this information must be corroborated.

#### Distribution.

Algeria, Angola, Brazil, Burkina Faso, Cameroon, Central African Republic, Democratic Republic of the Congo, Egypt, Ethiopia, Gambia, Ghana, Jamaica, Kenya, Liberia, Mali, Mexico, Mozambique, Namibia, Niger, Nigeria, South Africa, Saudi Arabia, Senegal, Sudan, Tanzania, Uganda, United Kingdom, Yemen, Zanzibar.

#### Discussion.

*Decellebruchus atrolineatus* has high economic importance, because it is a pest mainly in species in the genus *Vigna*. It is frequently found together with *Callosobruchus maculatus* (F.). Large losses due to this insect are reported frequently in some countries of Africa, where those bruchids are endemic (Booker 1967, Germain et al. 1987, Ofuya and Credland 1996, Sanon et al. 2005).

### Decellebruchus lunae

Taxon classificationAnimaliaColeopteraBruchidae

Romero sp. n.

http://zoobank.org/4156F64D-289A-4C0E-BA2A-18A7FBD80F10

#### Type series.

Holotype male, allotype female and two paratypes: M. Elgon, **Kenya**, 20/I/1979, 1900 m, Palm T. (MZLU), one paratype same data, except 22/I/1979, 2010 m (MZLU). One paratype: Kingsburg beach, Natal, **South Africa**, 10/IV/1992, O’Brien C.W., L.B. O’Brien & G. Marshall (CEAM).

#### Description.

**Figure 5.**
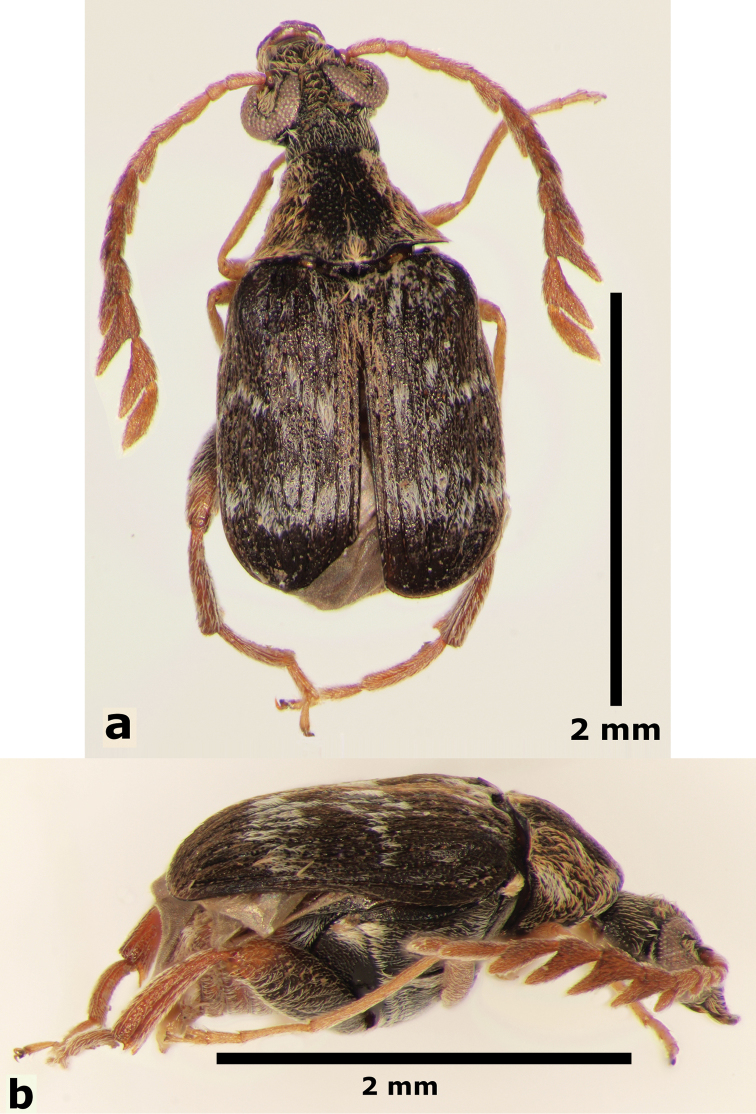
Male habitus of *Decellebruchus lunae*; **a** dorsal view **b** lateral view.

**Figure 6.**
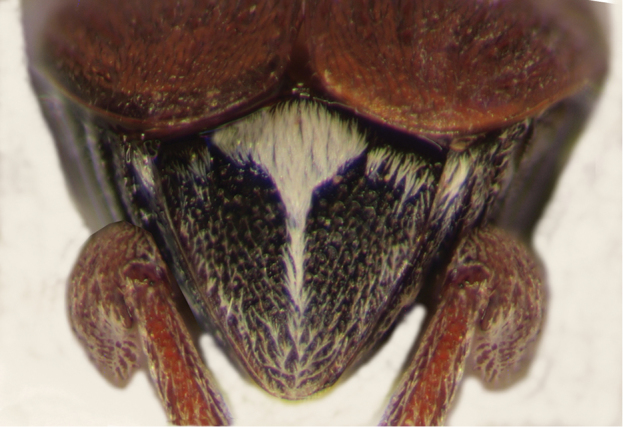
Male pygidium of *Decellebruchus lunae*.

**Figure 7.**
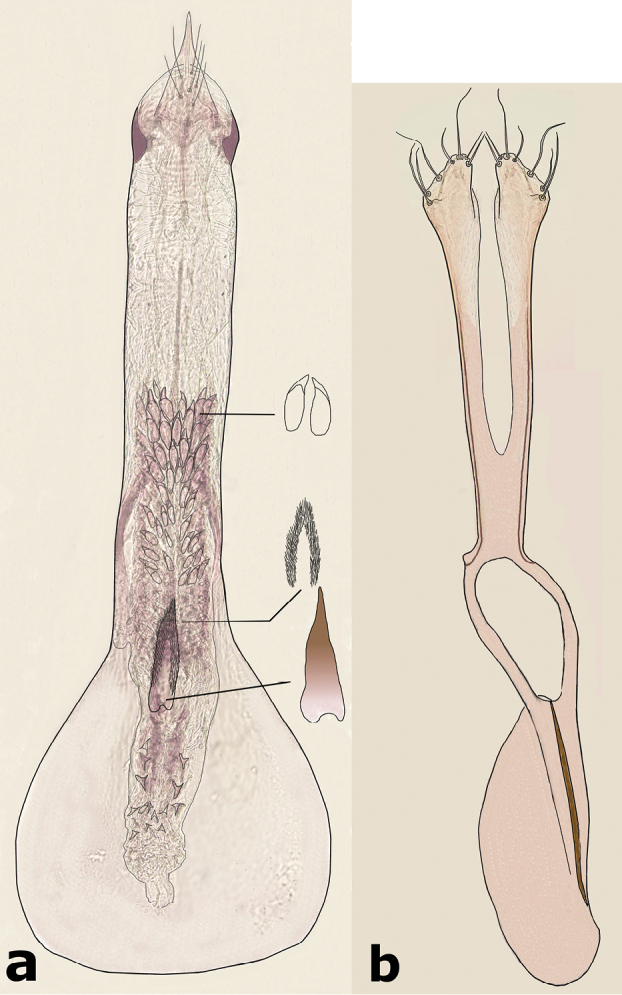
Male genitalia of *Decellebruchus lunae*; **a** median lobe **b** lateral lobes.

**Figure 8.**
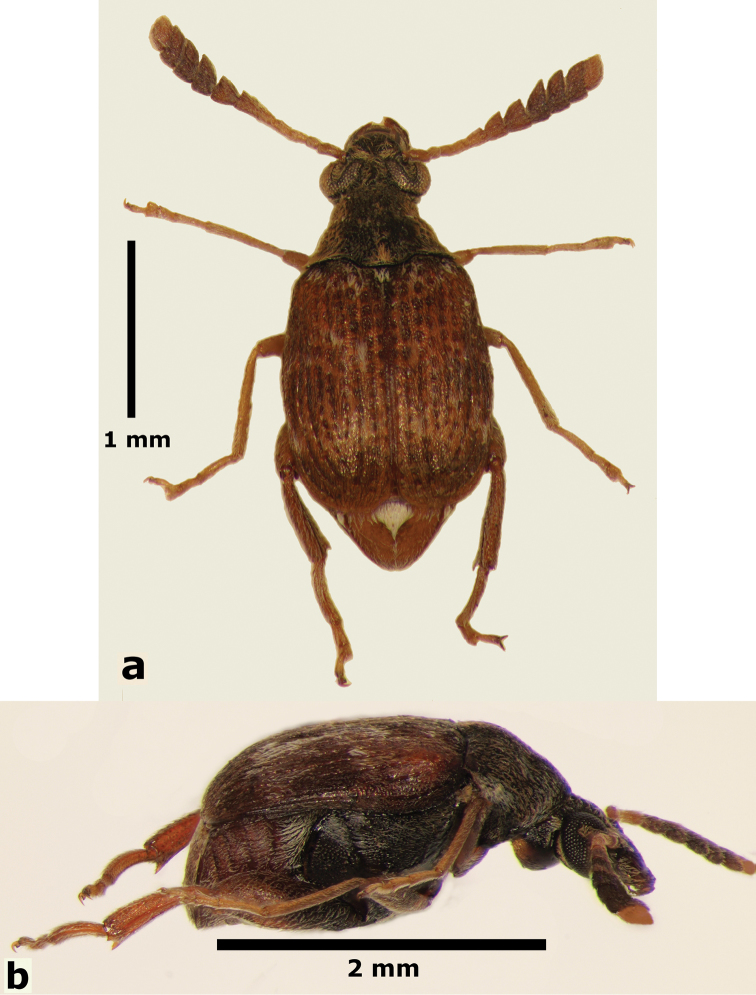
Female habitus of *Decellebruchus lunae*; **a** dorsal view **b** lateral view.

**Male** (Fig. 5a–b). Length (pronotum-elytra): 2.0–2.13 mm; width: 1.13–1.25 mm; maximum thoracic depth 1.1–1.2 mm. **Color**: Antennae with first four segments and apex of last one yellowish, the rest dark or partially dark; body dark, except fore legs, middle legs, elytra, tibiae and tarsi of posterior legs yellowish; however, some specimens may vary from all body yellowish to dark. **Vestiture**: Body with mixed black, yellowish, and white pubescence; fore coxa with a tuft of white setae; pygidium with basal central spot of white pubescence. **Head**: Short and broad, densely micropunctulate, frons with a strong median carina, distance between eyes 1.95–2.8× as wide as eye width, eye cleft 0.60–0.71× its length by ocular sinus, posterior margin of eye protruding from adjacent surfaces, postocular lobe rounded and setose; distance from base of antennae to apex of labrum 0.45–0.53× as long as distance from upper limits of eyes to apex of labrum; antennomeres I–III filiform, IV subserrate, V–XI pectinate; antennomere II 2–2.0× as long as antennomere 11; antennomere VII 1.76–2.0× wider that long; antenna extending to mid body. **Prothorax**: Subconical, without lateral carina; densely foveolate, disc convex, indistinctly gibbous before scutellum and without shallow median channel; prosternal process narrow, triangular, acute, half as long as procoxae. **Meso- and metathorax**: Scutellum square, bidentate apically; elytra with strial punctures wider than the stria, striae 4 and 5 abbreviated basally by tubercle, humeri slightly raised. **Legs**: First protarsomere 1.33–1.79× as long as second, first mesotarsomere 2.0–2.1× as long as second, first metatarsomere 2.6–3.2× as long as second; metacoxa densely punctate; hind femur constricted basally and apically, expanded medially to about width of coxa; without external carina ventrally; internal ventral carina with subapical acuminate spine about as long as width of tibial base; hind tibia straight, enlarged, with complete set of carinae; tibial corona with one spinule, the others obsolete, mucro 0.18–0.24× as long as first tarsomere; without sinus at base of spine; first hind tarsomere with ventrolateral glabrous longitudinal carina. **Abdomen**: Pygidium vertical (Fig. 6); last sternite emarginate. **Genitalia**: Median lobe moderately long, ventral valve triangular and deeply arcuate, internal sac lined with many small spines, basal portion with a dentiform sclerite and a small spinules forming a triangle (Fig. 7a); lateral lobes elongate, expanded at apex, cleft about 0.73 their length; basal strut with a strong perpendicular keel (Fig. 7b). **Female** (Fig. 8a–b). Length (pronotum-elytra) 1.85–2.05 mm, width: 1.12–1.3 mm, Maximum thoracic depth 0.95–1.41 mm. Similar to male except antennae serrate; pygidium subvertical; last sternite not marginate.

#### Host plant.

Unknown.

#### Distribution.

Kenya and South Africa.

#### Etymology.

The specific epithet honors the grandaughter of the author, Luna Nereida Nila Romero.

#### Diagnosis.

This species is included in the genus *Decellebruchus* because it presents all characters indicated in the diagnosis of the genus; also it can be separated from the other two species in the genus because the typical male pygidium, less strongly male antennae, unique armature of the internal sac of male genitalia, and lateral lobes of which bear a basal strut with a strong perpendicular keel.

### Decellebruchus walkeri

Taxon classificationAnimaliaColeopteraBruchidae

(Pic, 1912)

Bruchus figuratus Walker, 1859: 261 (homonymy); Pic 1913: 57; Decelle 1975b: 184; Vazirani 1975: 746; Decelle 1985: 75.
Bruchus walkeri Pic, 1912: 92 (replacement name), 1913: 57; Decelle 1975b: 184; Vazirani 1975: 746; Decelle 1985: 76.
Bruchidius walkeri : Decelle 1975b: 184; Decelle 1985: 75.
Spermophagus figuratus : Motschulsky 1863: 519; Decelle 1975b: 185;

#### Description.

**Figure 9.**
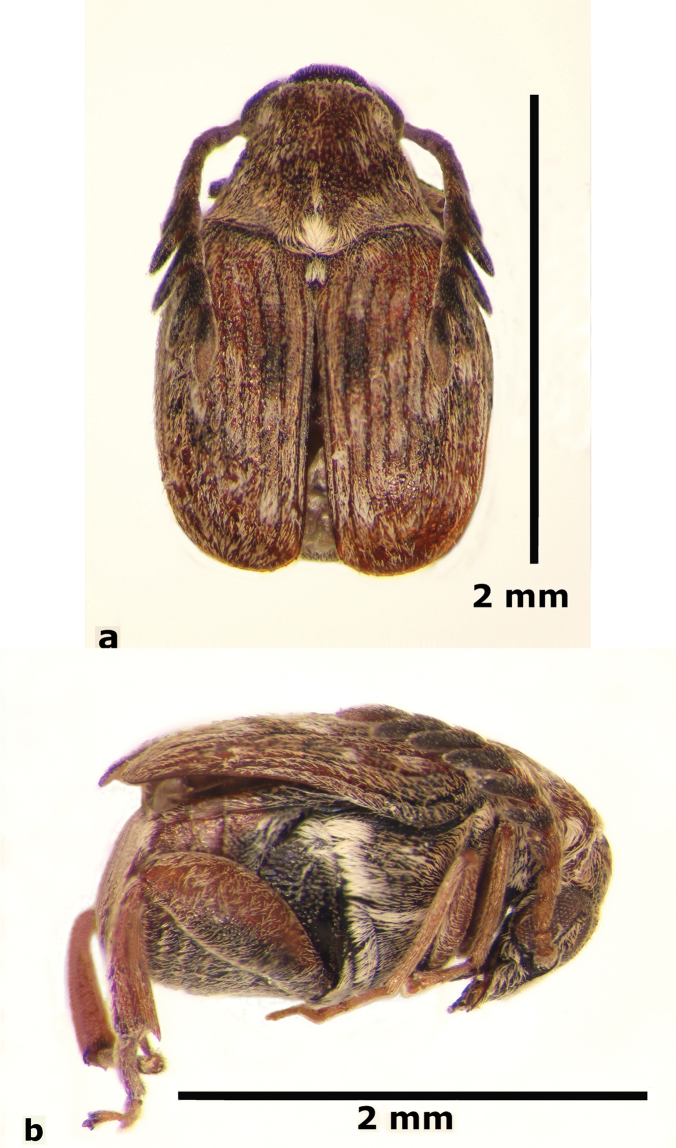
Male habitus of *Decellebruchus walkeri*; **a** dorsal view **b** lateral view.

**Figure 10.**
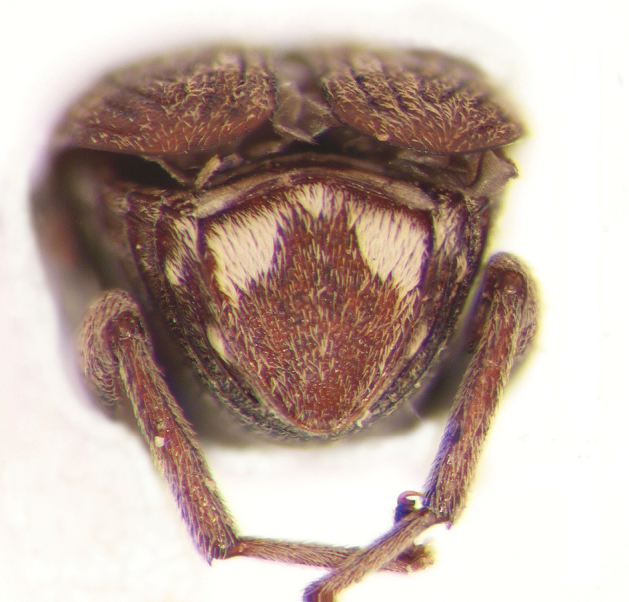
Male pygidium of *Decellebruchus walkeri*.

**Figure 11.**
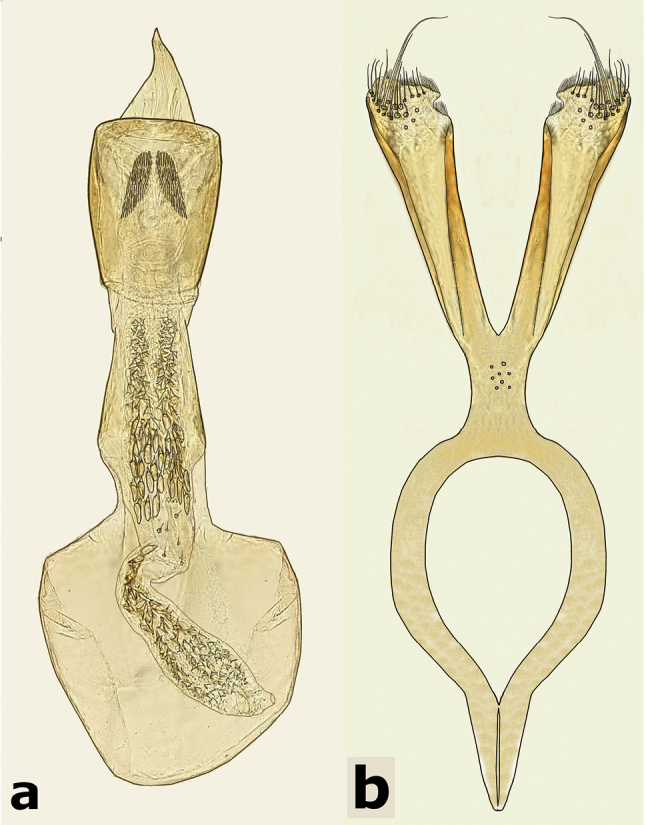
Male genitalia of *Decellebruchus walkeri*; **a** median lobe **b** lateral lobes.

**Figure 12.**
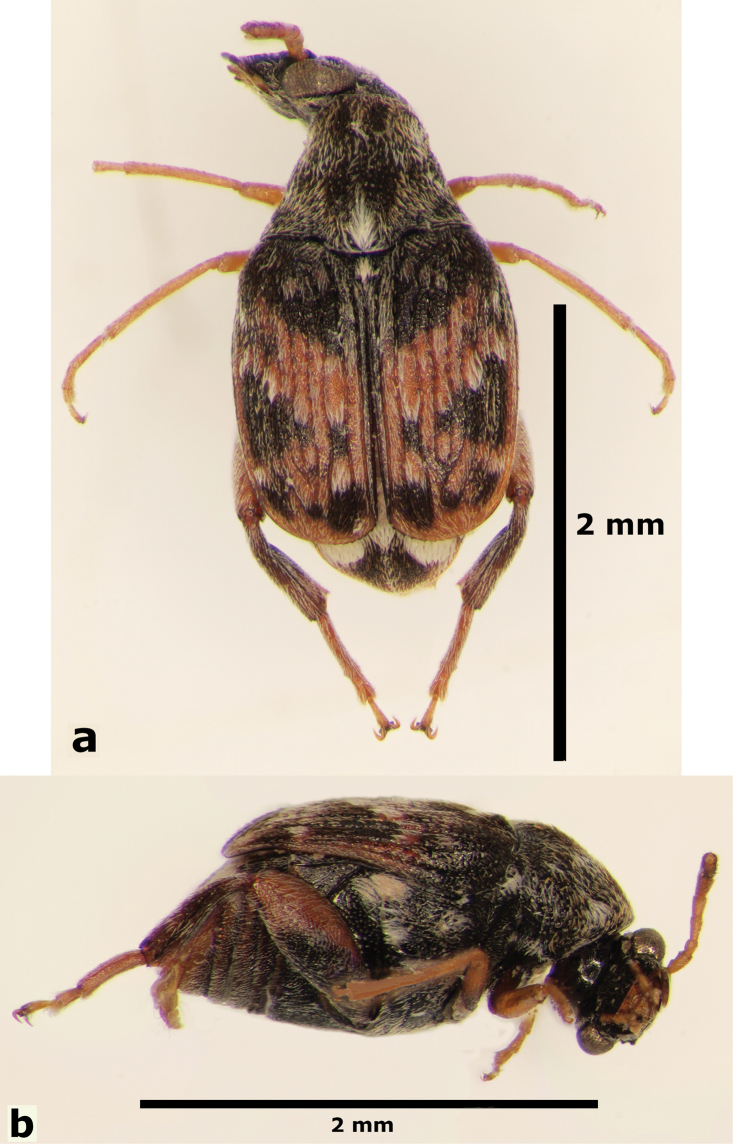
Female habitus of *Decellebruchus walkeri*; **a** dorsal view **b** lateral view.

**Male** (Fig. 9a–b). Length (pronotum-elytra): 1.77–2.1 mm; width: 1.27–1.37 mm; maximum thoracic depth 1.07–1.32 mm. **Color**: Antennae with the first five segments and apex of the last one yellowish, the rest dark or partially dark; body dark, except fore legs, middle legs, part of elytra, and ventral portion of femora; however some specimens may vary from all body yellowish to dark. **Vestiture**: Body with mixed black, yellowish, and white pubescence; fore coxa with a tuft of white setae; pygidium with three basal spots of white pubescence, the lateral ones bigger than median. **Head**: Short and broad, densely micropunctulate, frons with a strong median carina, distance between eyes 1.53–3.3× as wide as eye width, eye cleft 0.57–0.8× its length by ocular sinus, posterior margin of eye protruding from adjacent surfaces, postocular lobe rounded and setose; distance from base of antennae to apex of labrum 0.38–0.47× as long as distance from upper limits of eyes to apex of labrum; antennomeres I–III filiform, IV subserrate, V–XI pectinate; antennomere II 2–2.0× as long as antennomere 11; antennomere VII 4.75–6.67× wider that long; antenna extending to mid body. **Prothorax**: Subconical, without lateral carina; densely foveolate, disc convex, lightly gibbous before scutellum and without shallow median channel; prosternal process narrow, triangular, acute, half as long as procoxa. **Meso- and metathorax**: Scutellum square, bidentate apically; elytra with striael punctures as wide as the stria, striae 4 and 5 abbreviated basally by tubercle, humeri slightly raised. **Legs**: First protarsomere 1.45–1.7× as long as second, first mesotarsomere 1.92–2.6× as long as second, first metatarsomere 2.86–3.4× as long as second; metacoxa densely punctate; hind femur constricted basally and apically, expanded medially to about width of metacoxa; without external carina ventrally; internal ventral carina with subapical acuminate spine about as long as width of tibial base, followed by 2 smaller spines; hind tibia straight, enlarged, with complete set of carinae; tibial corona with 4 spinules, mucro 0.11–0.16× as long as first tarsomere; without sinus at base of spine; first hind tarsomere with ventrolateral glabrous longitudinal carina. **Abdomen**: Pygidium vertical (Fig. 10); last sternite emarginate. **Genitalia**: Median lobe moderately long, ventral valve triangular and deeply arcuate, internal sac lined with many small spines, basal portion with a small spinules forming a triangle (Fig. 11a); lateral lobes elongate, expanded at apex, cleft about 0.66 their length; basal strut with small perpendicular keel (Fig. 11b). **Female** (Fig. 12a–b). Length (pronotum-elytra) 1.95–2.8 mm, width: 1.25–1.8 mm, Maximum thoracic depth 1.12 mm. Similar to male except antennae serrate; pygidium subvertical; last sternite not marginate.

#### Material examined.

INDIA: Maharashtra, Lonavla, 28/IV/2000, 650 m, Pacholatko P. (1 ex., CEAM). KENYA: M. Elgon, 24/I/1979, 1950 m, Palm T. (1 ex., MZLU). SRI LANKA: Vayiriuttu, 5 mi W Trincomalee, Estern Prov., 9/II/1962, Lund University Ceylon Expedition, sweeping at teak plantation (1 ex., MZLU); Kuda Oya, 15 mi S Wellawaya, Uva Prov., 22/III/1962, Brinck, Andersson & Cederholm (2 ex., MZLU); Yakkala, 18 mi NE Colombo Western Prov., 26/III/1926, Brinck, Andersson & Cederholm, swept on vegetation at ditches in paddy fields (1 ex., MZLU).

#### Host plant.

Unknown.

#### Distribution.

India, Kenya, Sri Lanka, Thailand.

#### Discussion.

There is little information about this species. At this time its host plants are unknown and only a few specimens were available for study; three of which were still named *Bruchidius walkeri*.

## Cladistics

**Figure 13.**
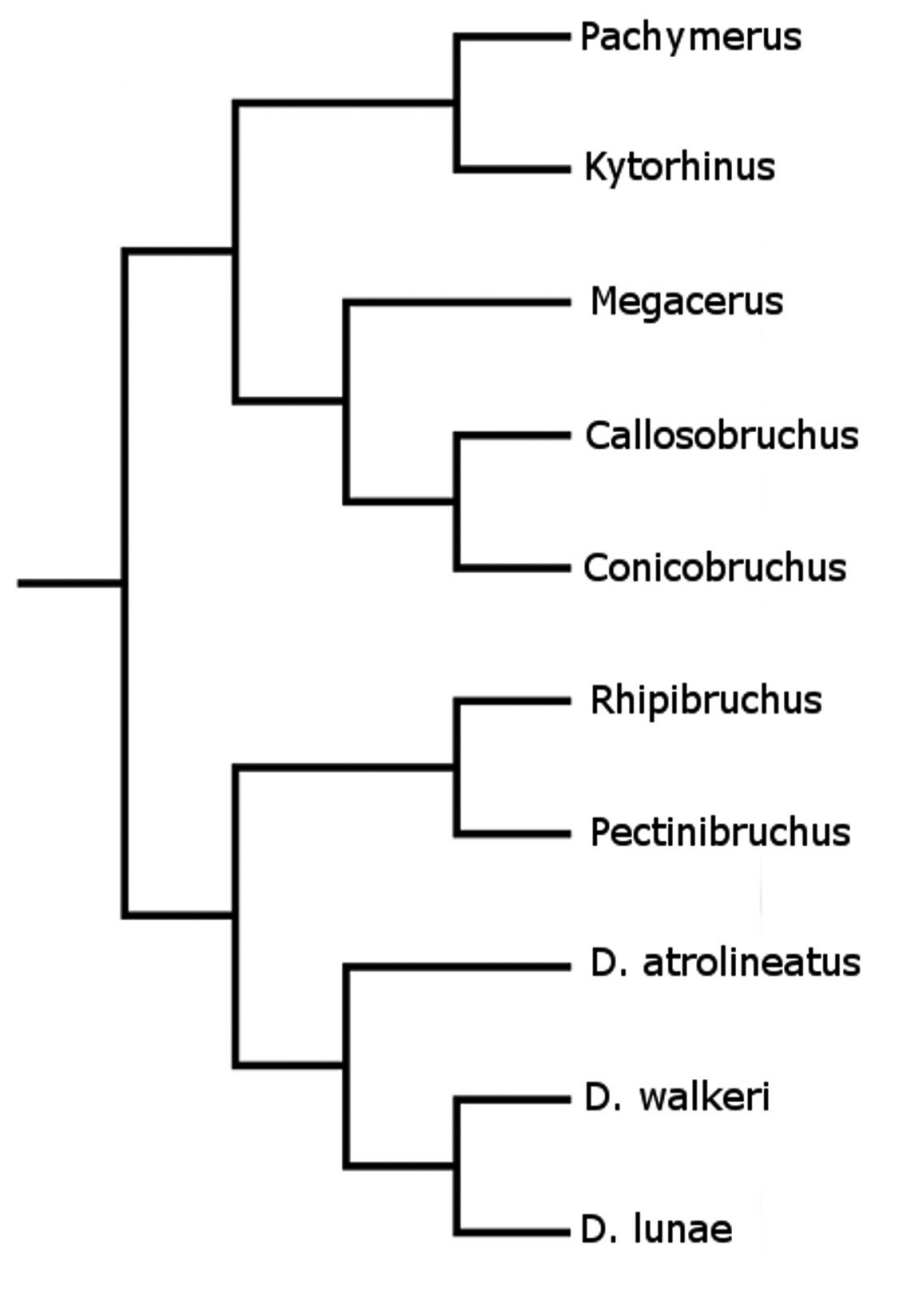
Default tree generated with Mesquite program.

**Figure 14.**
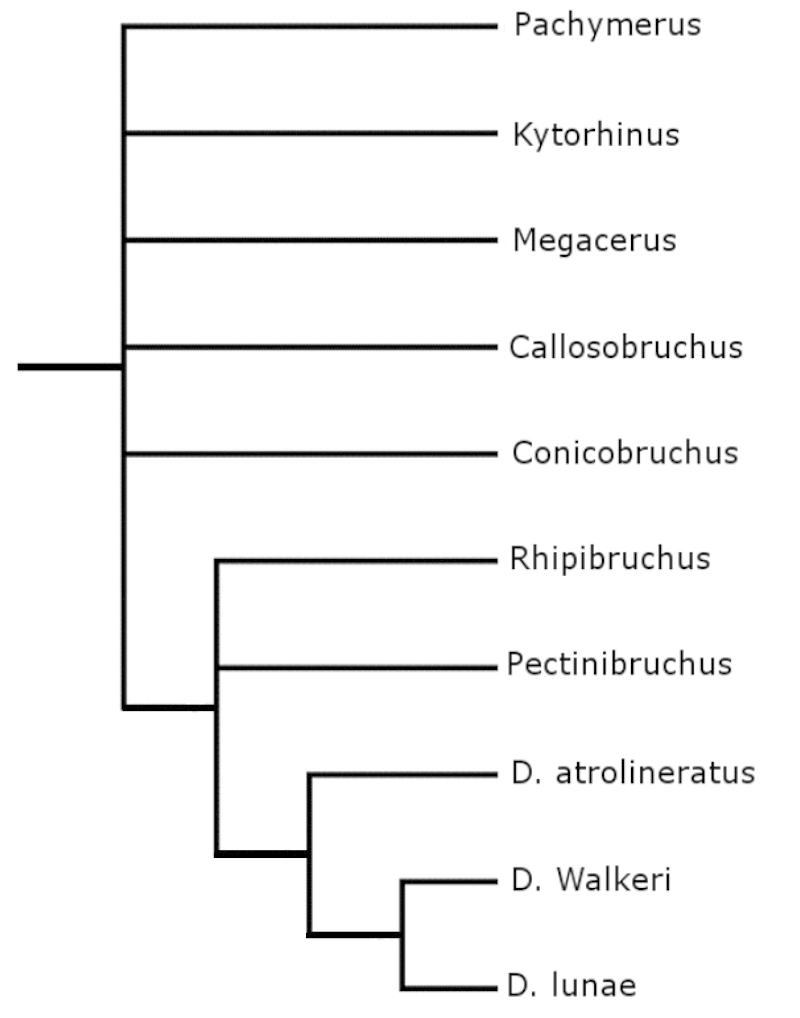
Consensus tree generated with Mesquite program.

**Figure 15.**
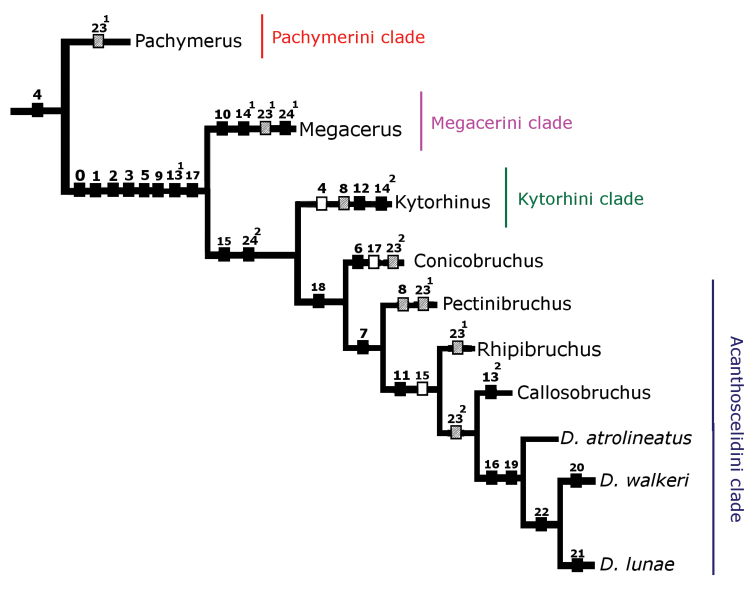
Phylogenetic tree generated with Hennig86 program.

A default tree (Fig. 13) and a consensus tree (Fig. 14) were generated with Mesquite (Maddison and Maddison 2015). The shortest and most parsimonius tree obtained with Henning86 using ie- algorithm is shown in Fig. 15. This tree was the shortest and the most parsimonious with a length of 33, consistency index of 87, and retention index of 81. In total, this cladogram was formed by 26 synapomorphies, 8 parallelisms, and 3 reversals. The tree generated with Hennig86 seems the most reliable to explain the phylogenetic hypothesis about of bruchids with pectinate antennae where males and females share the character; however, the consensus tree generated with Mesquite program has similarities with the Hennig86 tree.

In the cladogram in Figure 15 can be seen that each one of the clades corresponds to a different taxon of the family Bruchidae and one of the richest in terms of the number of genera was the Acanthoscelidini clade. This cladogram also supports the hypothesis of host preference, for example the species of the clade Pachymerini feed on seeds of the Arecaceae (palms), Megacerini on seeds of the Convolvulaceae, and Acanthoscelidini on seeds of the Fabaceae.

## Supplementary Material

XML Treatment for Decellebruchus

XML Treatment for Decellebruchus atrolineatus

XML Treatment for Decellebruchus lunae

XML Treatment for Decellebruchus walkeri
